# COVID-GWAB: A Web-Based Prediction of COVID-19 Host Genes via Network Boosting of Genome-Wide Association Data

**DOI:** 10.3390/biom12101446

**Published:** 2022-10-09

**Authors:** Seungbyn Baek, Sunmo Yang, Insuk Lee

**Affiliations:** 1 Department of Biotechnology, College of Life Science and Biotechnology, Yonsei University, Seoul 03722, Republic of Korea; 2 POSTECH Biotech Center, Pohang University of Science and Technology (POSTECH), Pohang 37673, Republic of Korea

**Keywords:** COVID-19, genome-wide association study, network boosting

## Abstract

Host genetics affect both the susceptibility and response to viral infection. Searching for host genes that contribute to COVID-19, the Host Genetics Initiative (HGI) was formed to investigate the genetic factors involved in COVID-19 via genome-wide association studies (GWAS). The GWAS suffer from limited statistical power and in general, only a few genes can pass the conventional significance thresholds. This statistical limitation may be overcome by boosting weak association signals through integrating independent functional information such as molecular interactions. Additionally, the boosted results can be evaluated by various independent data for further connections to COVID-19. We present COVID-GWAB, a web-based tool to boost original GWAS signals from COVID-19 patients by taking the signals of the interactome neighbors. COVID-GWAB takes summary statistics from the COVID-19 HGI or user input data and reprioritizes candidate host genes for COVID-19 using HumanNet, a co-functional human gene network. The current version of COVID-GWAB provides the pre-processed data of releases 5, 6, and 7 of the HGI. Additionally, COVID-GWAB provides web interfaces for a summary of augmented GWAS signals, prediction evaluations by appearance frequency in COVID-19 literature, single-cell transcriptome data, and associated pathways. The web server also enables browsing the candidate gene networks.

## 1. Introduction

The global outbreak of coronavirus disease 2019 (COVID-19), a disease caused by SARS-CoV-2, became a pandemic that affected numerous people worldwide [1]. Although patients with COVID-19 predominantly suffer from symptoms related to the respiratory system, the degree of disease severity and progression has been heterogeneous, ranging from asymptomatic to lethal conditions [2]. Due to its complexity in disease responses, there have been worldwide efforts to understand the various factors that influence COVID-19 symptoms [3]. Among those COVID-19-related factors, host genetics greatly affect the disease’s initiation and progression [4].

Host genetics affect the susceptibility and response to viral infection. Searching for host genes contributing to COVID-19, researchers formed a global network to investigate the host genetic factors involved in COVID-19 via genome-wide association studies (GWAS) (https://www.covid19hg.org/ (accessed on 22 June 2022)). The initial publication was based on 49,562 COVID-19 patients [4]. Since then, the cohort size has continued to increase, and the latest published release of GWAS summary statistics is based on 125,584 patients [5]. GWAS suffer from limited statistical power and generally, only a few genes can pass the conventional significance thresholds (e.g., *p* ≤ 5 × 10^−8^). Accordingly, the published study with 49,562 patients reported only 13 human genome loci for COVID-19 [4]. This statistical limitation may be overcome by augmenting weak association signals through integrating independent functional information such as molecular interactions [6].

Human gene networks are composed of numerous molecular interactions that can be used to explain the complexity of human diseases. Because diseases are commonly associated with dysfunctions in several pathways, identifying disease-related genes and their co-functional genes connected within the networks can expand our knowledge of diseases. Therefore, network-based analysis of GWAS data can lead to valuable discoveries. For network-based GWAS analysis, the first method is the identification of subnetworks. The candidate genes from GWAS are assigned scores based on their *p* values of GWAS significance and mapped back to the networks for identifying the disease-related subnetworks. Those subnetworks could be composed of pathways and gene interactions that are essential for diseases [7,8]. The second method is the reprioritization of candidate genes connected through the networks. GWAS associations of the co-functional genes are boosted by signals from nearby neighbors, which can lead to the identification of new candidate genes with sub-threshold disease associations with GWAS alone [6,9,10]. In addition to using networks, the evaluation of the new candidate genes with other resources, such as a collection of experimental evidence, transcriptome datasets, and biological pathways, can further validate disease relevance.

Here, we present COVID-GWAB (https://inetbio.org/covidgwab/ (accessed on 2 September 2022)), a web-based tool for boosting the original GWAS signals of individual genes for COVID-19 by integrating those of their interactome neighbors and comparing the results with various other datasets and literature sources. COVID-GWAB provides simple step-by-step web interfaces that can initiate network-based boosting of the GWAS data, understand the boosted results and provide a summary, and further validate and discover additional resources. With COVID-GWAB, researchers can find new COVID-19-related candidate genes with GWAS data.

## 2. Materials and Methods

### 2.1. GWAS Data Sources and the Human Gene Network

The COVID-GWAB server takes summary statistics data from the COVID-19 Host Genetics Initiative (HGI) or user input GWAS data. The current version of COVID-GWAB provides the pre-processed data of Releases 5, 6, and 7 of the COVID-19 HGI GWAS data (https://www.covid19hg.org/ (accessed on 22 June 2022)). We will continue to update the server with any future releases of the COVID-19 HGI GWAS data. The COVID-19 HGI GWAS data are composed of four different phenotype comparison results: A2 (very severe respiratory confirmed COVID-19 vs. population), B1 (hospitalized COVID-19 vs. not hospitalized COVID-19), B2 (hospitalized COVID-19 vs. population), and C2 (COVID-19 vs. population). COVID-GWAB uses a human gene network, HumanNet (version 3) [11]. Of the three-tier models of the network, the most conservative model, HumanNet-PI, composed of only protein–protein interactions, is used for the network-based boosting of GWAS data.

### 2.2. COVID-19 Host Gene Predictions by Network-Based Boosting

**Figure 1:**
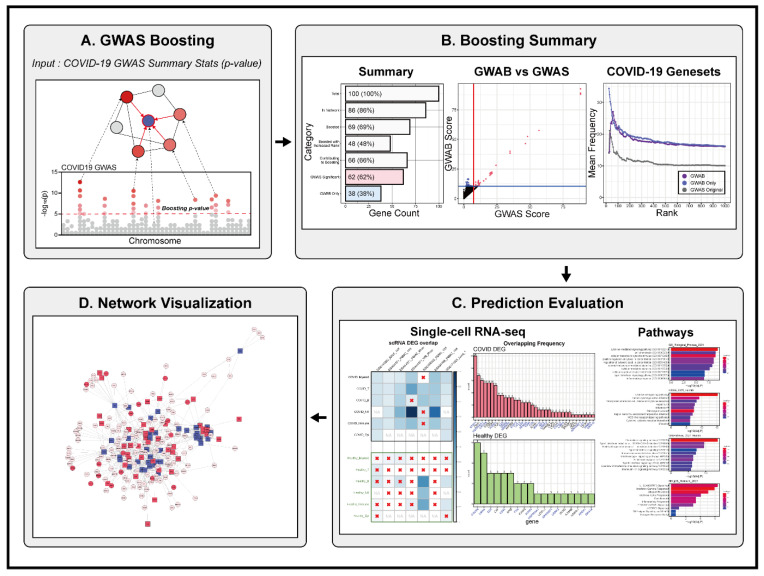
Overview of COVID-GWAB. (**A**) Graphical summary of the method for the network boosting of GWAS data, (**B**–**D**) Representative web interfaces for summary reports of the network boosting (**B**), prediction evaluation (**C**), and visualizing a candidate gene network (**D**). A red × in (**C**) indicates no significant overlap for the corresponding categories (*p* > 0.05, Fisher’s exact test).

COVID-GWAB conducts the network-based boosting of GWAS data proposed in our previous works [6,12]. To augment the GWAS signals using a gene network, we first assign the *p* values of SNPs to genes within a designated chromosomal distance by user input (Figure 1A). If multiple *p* values are assigned to a given gene, the best *p*-value is considered for the downstream analysis. For the network-based boosting of GWAS data, we implemented the scoring scheme described in our previous works [6,12]. To acquire information from the genes close to being statistically significant, we used a ‘soft’ guilt-by-association (GBA) by $\left(p_{j}-\left(1-p_{j}\right)\right)$, in which $p_{j}$ is a probability of disease involvement of a gene *j*. With the soft GBA, genes with strong disease associations would be given full weight. For the network neighboring gene *j* of gene *i*, the total contributions of the GWAS association scores are calculated using the following equation:

$$S_{i}=\underset{j}{\sum }\left(2p_{j}-1\right)l_{ij}$$

in which $l_{ij}$ is the likelihood score of the link between gene *i* and gene *j* in the co-functional network. We calculated the likelihood score of the links based on a Bayesian statistics framework in which the ability to capture known links is evaluated for the given standards [13]. We then integrated the GWAS data into the co-functional network in a naïve Bayes framework, given that the data from each of them were conditionally independent. We calculated the GWAB scores, the posterior log odds that gene *i* is involved in the disease, using the following equation:

$$\log O\left(i\in D|D_{Net}D_{GWAS}\right)=S_{i}+\log O(i\in D|D_{GWAS})$$

in which $\log O\left(i\in D|D_{Net}D_{GWAS}\right)$ is the log odds of the association calculated from the GWAS data, which is equal to the log Bayes factor for the disease association added by the prior log odds for the association. The *p* values from the GWAS data were used for the calculation of the odds of the association. We excluded genes encoding the major histocompatibility complex (MHC) molecules from the final candidates because their inflated associations with COVID-19 driven by the unusual genomic structures of MHC regions have been reported [14,15,16].

### 2.3. Web Interfaces for Facilitating the Interpretation of the Boosting Results

COVID-GWAB provides web interfaces for summarizing the boosting results, prediction evaluations, and a candidate gene network (Figure 1B–D). To evaluate the effectiveness of the network boosting, we compared the predictions of COVID-GWAB with those of GWAS alone. The relevance of the candidate genes with COVID-19 was estimated by the frequencies of appearances of each gene in all COVID-19-related studies that were summarized by The COVID-19 Drug and Gene Set Library [17]. For the validation of the predictions, we collected COVID-19 single-cell transcriptomics datasets from five independent studies [18,19,20,21,22] (Table S1). All cell types were based on annotations from the original articles, and differentially expressed genes (DEGs) were calculated by comparing COVID-19 patient samples to healthy controls with Seurat’s FindMarkers functions (ln(FoldChange) > 0.25 & adjusted *p* value < 0.01) [23]. We calculated the overlaps between the GWAB results and DEGs from the single-cell datasets using Fisher’s exact test. We conducted the pathway analysis using enrichR [24]. All the plots for the Boosting Summary and Prediction Evaluation sections were drawn using ggplot2 [25]. We constructed the gene network using the GWAB results by extracting the subnetworks from HumanNet [11] of the GWAB result genes and adjacent nodes to the GWAB result genes. The centrality scores were measured with igraph’s betweenness and degree functions [26]. The web server can also visualize the network of candidate genes with centrality scores, COVID-19 gene set library frequencies, and rank changes from the GWAS summary statistics *p* values to GWAB scores after the network boosting. Users can easily browse the pre-calculated COVID-GWAB results using the COVID-19 HGI GWAS data or run their datasets with various parameters, such as SNP distances to genes and GWAS *p* value thresholds, for the network boosting.

## 3. Results

### 3.1. Comparison of GWAB and GWAS-Only Results Using COVID-19 Geneset Library

**Figure 2:**
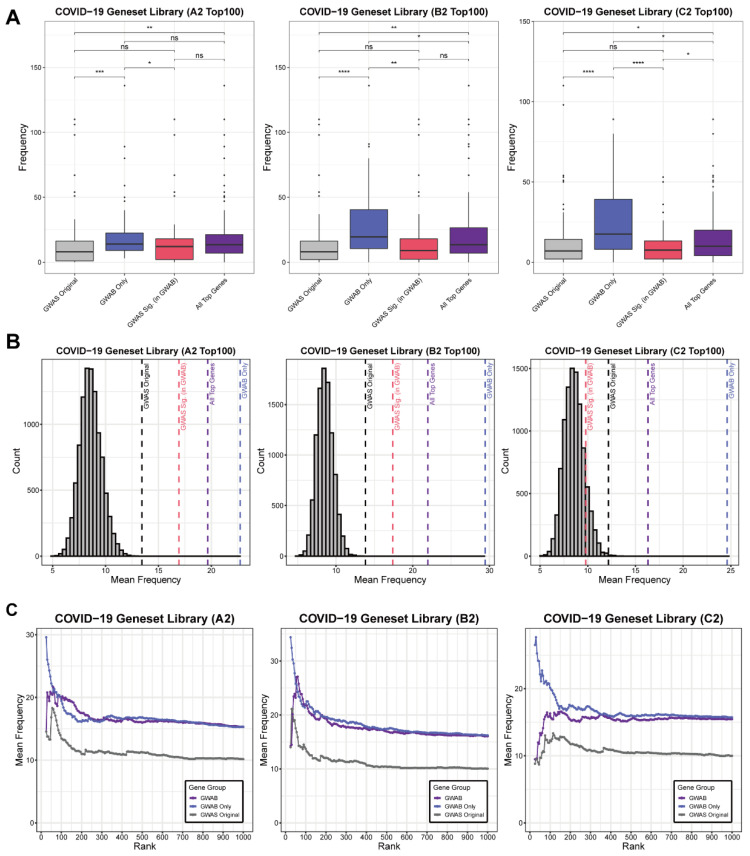
Frequency of appearance in COVID-19 gene set library. Release 6 of COVID-19 HGI GWAS data; A2, B2, and C2 are used. GWAS Originals are top GWAS results based on GWAS statistics *p* values without boosting. GWAB Onlys are genes in GWAB Top N results (ranked with GWAB scores) that have GWAS statistics *p* values bigger (not significant) than 5 × 10^−8^. GWAS Sigs are genes in GWAB Top N results with GWAS statistics *p* values smaller (significant) than 5 × 10^−8^. All Top Genes are genes in GWAB Top N results. (**A**) Comparison of frequencies of GWAB top 100 genes for each gene group. The Wilcoxon signed-rank test is used for the *p* values. Statistical significance: ns (*p* > 0.05), * (*p* ≤ 0.05), ** (*p* ≤ 0.01), *** (*p* ≤ 0.001), **** (*p* ≤ 0.0001) (**B**) Histogram with mean COVID-19 geneset library frequencies of 100 random genes from all genes, repeated 10,000 times. Each dashed line indicates the mean frequency of each gene group. (**C**) Mean frequencies of top-ranked genes from each gene group ranked from 25 to 1000 (increased by 5).

We used Release 6 of the COVID-19 HGI GWAS data to compare the predictions from COVID-GWAB to the top predictions by GWAS alone. The top results for GWAS alone were defined by the *p* values from the GWAS summary statistics and the top results for COVID-GWAB were defined by the GWAB scores. As with publications from the COVID-19 HGI, we used phenotypes A2, B2, and C2, and excluded phenotype B1 (hospitalized COVID-19 vs. not hospitalized COVID-19). In order to survey the biological significance and relevance of the genes from each category, we used the COVID-19 geneset library [17], which collected COVID-19-related studies to generate the appearance frequency count for each gene from those studies. We compared the top 100 results from GWAS alone (GWAS Original), the top 100 results from COVID-GWAB (All Top Genes), and the new candidate genes (GWAB Only) from the top 100 COVID-GWAB results without GWAS significance (*p* < 5 × 10^−8^) and GWAS-significant genes in the top 100 COVID-GWAB results. Across all three COVID-19 GWAS phenotypes, “GWAB Only” showed the highest frequency followed by “All Top Genes”, “GWAS Sig.”, and “GWAS Original” (Figure 2A). Furthermore, we randomly selected 100 genes from the COVID-GWAB results and calculated their mean frequency. We repeated this process 10,000 times to generate a random gene frequency distribution. All four gene categories showed higher mean frequencies than the random distribution, indicating their significant connections to various COVID-19 experimental results. As with the previous results, the “GWAB only” category showed the highest mean frequency (Figure 2B). The overall trend in the mean frequencies for the top 25 to 1000 genes with “All Top Genes”, “GWAS Only”, and “GWAS Original” results showed consistently larger frequencies after the network-based boosting (Figure 2C). Therefore, these results show that although GWAS results alone still have a biological connection to COVID-19-related experiments and literature, COVID-GWAB better captures these relationships by incorporating human functional gene networks for more interpretable candidate genes.

### 3.2. Comparison of GWAB and GWAS Alone Results Using COVID-19 Single-Cell RNA-seq Datasets

**Figure 3:**
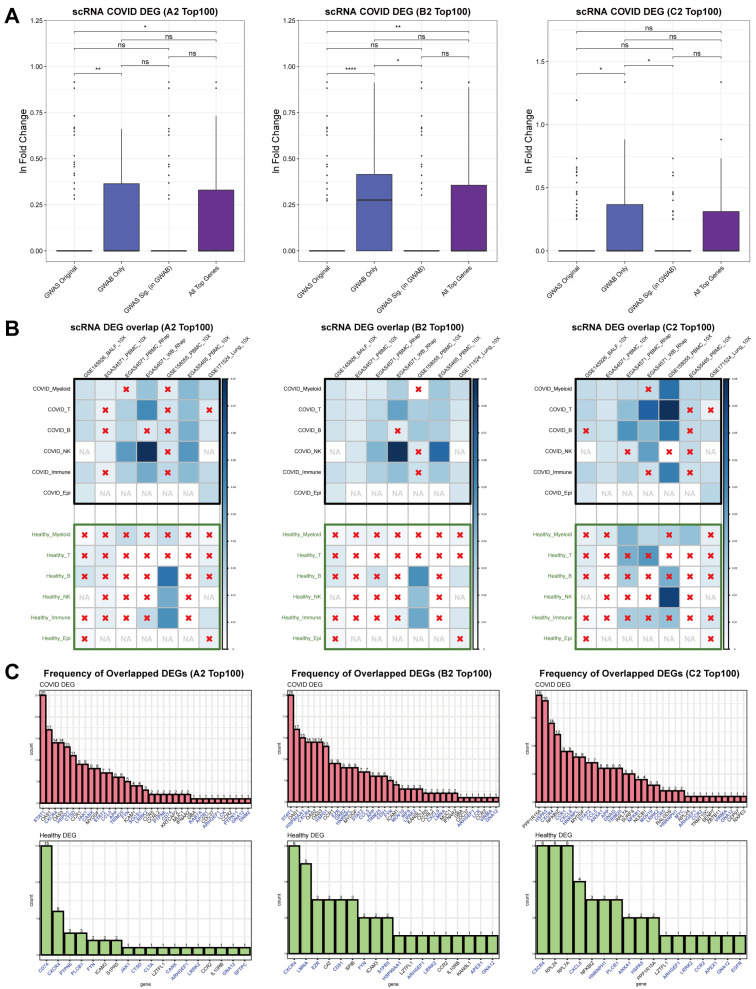
Overlaps of differentially expressed genes (DEGs) between COVID patients and healthy controls for each cell type from single-cell RNA-seq datasets. (**A**) Comparison of fold changes of GWAB top 100 genes for each gene group. The Wilcoxon signed-rank test is used for the *p* values. Statistical significance: ns (*p* > 0.05), * (*p* ≤ 0.05), ** (*p* ≤ 0.01), **** (*p* ≤ 0.0001). (**B**) Overlaps between DEGs and GWAB top 100 genes. The color scale indicates the overlap percentage, a red × indicates no significant overlap for the corresponding categories (*p* > 0.05, Fisher’s exact test), and NA indicates no available DEGs for the corresponding categories. (**C**) Overlap counts for COVID DEGs and control DEGs. The genes in blue are the ‘GWAB Only’ genes.

To further compare biological relevance and validate the COVID-GWAB results, we utilized various single-cell RNA sequencing datasets comparing COVID-19 patients and healthy controls. The datasets covered various regional cohorts and tissue types such as peripheral blood mononuclear cells (PBMCs), whole blood, bronchoalveolar lavage fluid (BALF), and lung (Table S1). We calculated the differentially expressed genes (DEGs) for COVID-19 patients and healthy controls for each dataset. Using the same gene categories as above, we compared the fold changes of the genes from all cell types and datasets combined. The results showed that the “GWAB Only” results had higher fold change values toward COVID-19 patients compared to “GWAS Original” (Figure 3A). We used Fisher’s exact test and overlap percentages to analyze cell-type and disease-specific overlaps between the top results from COVID-GWAB and the DEGs from the single-cell datasets. Figure 3B shows overall more significant overlap counts for the DEGs from COVID-19 patients for most datasets without cell-type specific enrichment. Next, we counted the actual overlap counts for genes in the top 100 COVID-GWAB results. The top genes showed more overlap counts for the COVID-19 DEGs and more than half of the genes with overlap counts were categorized as “GWAB Only” genes (Figure 3C). Overall, newly found candidate genes with COVID-GWAB showed significantly higher log fold changes and overlap counts with the COVID-19-related genes calculated from actual patient datasets. Furthermore, those top genes were more enriched for COVID-19 patients than healthy controls, which indicates COVID-GWAB’s ability to boost GWAS statistics in a disease-relevant manner.

### 3.3. Validation of GWAB Candidates by Literature Survey

**Figure 4:**
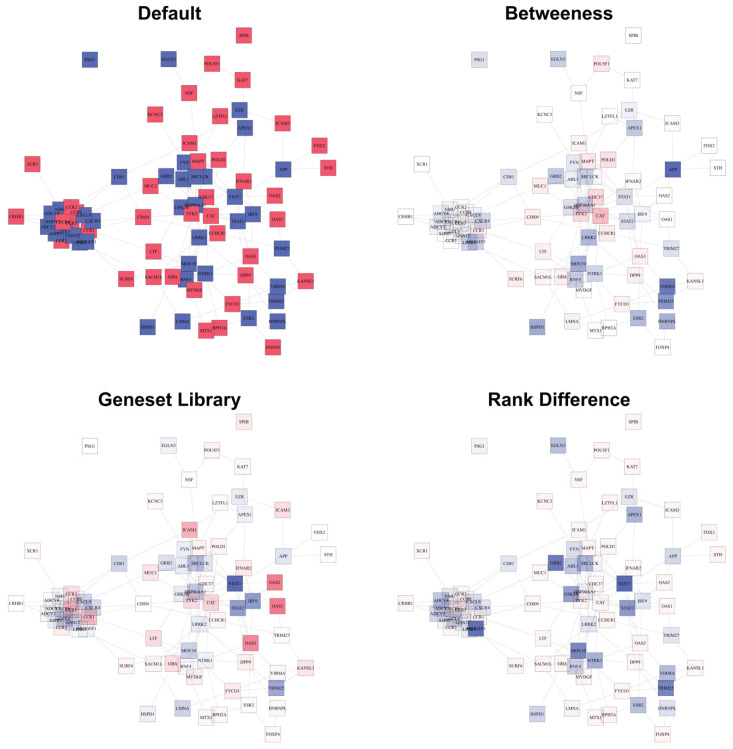
Network visualization of top 100 GWAB-COVID results with B2 of HGI GWAS Release 6. Only genes included in HumanNet are included in this network. Adjacent genes that are visualized in the web server are excluded here for simplification. For the “Default” network graph, the blue nodes are the newly discovered genes that are not GWAS-significant. The red nodes are the GWAS significant genes. For the rest of the network graphs, the nodes have more transparency with lower ranks based on their betweenness centrality, COVID-19 geneset library frequency, and rank changes from GWAS alone to COVID-GWAB.

To demonstrate the feasibility of the identification of novel host genes for COVID-19 by the network boosting of GWAS data, we submitted the GWAS summary statistics data from phenotype B2 from Release 6 of the COVID-19 HGI. We examined the candidate genes that could not have been suggested by GWAS alone via manual literature surveys (Table S2). Furthermore, those top genes are visualized as connected networks and colored accordingly to their betweenness centrality, appearance frequency in the COVID-19 geneset library, and rank change after COVID-GWAB boosting (Figure 4). Chemokine receptors, such as *CCR9, CXCR6, CCR1, CCR3, CCR5*, and *CCR2*, are all located on chromosome 3 and already showed significant associations with COVID-19 without network boosting. Other chemokines and chemokine receptors became significant candidates only after boosting (GWAB Only). For example, the priority ranks substantially increased from GWAS alone to GWAB (denoted as GWAS rank → GWAB rank in the following) for *CXCR4* (2337th → 42nd), *CCL5* (5142nd → 43rd), and *CXCL9* (3981st →96th), and they were found to be involved in various stages of SARS-CoV-2 infection [27]. Several GWAB-only candidates such as *EGFR* (17,798th →134th), *ANXA1* (12,269th → 302nd), *HNRNPL* (3116th → 21st), *MOV10* (16,917th → 41st), and *STAT2* (14,012th →38th) were recently found to interact with the SARS-CoV-2 RNA in infected human cells [28]. *TRIM25* (17,506th → 34th) is known to be involved in antiviral innate immunity and SARS-CoV-1 [29,30,31]. Intriguingly, *APP* (4621st → 24th) and *LRRK2* (2629th → 25th) that are involved in Alzheimer’s disease and Parkinson’s disease, respectively, were suggested as highly probable candidates by GWAB. Multiple studies recently suggested the possible connection between such neurodegenerative diseases and COVID-19 [32,33,34,35,36]. Several kinases, such as *NTRK1* (10,495th → 29th), *FYN* (3902nd → 46th), *ABL1* (7261st → 91st), and *SRC* (8965th → 56th), are being studied for repurposing several kinase inhibitors for COVID-19 treatment [37,38]. Furthermore, *STAT1* (21,435th → 32nd), *STAT2, EGFR*, and *IRF9* (2455th → 36th) are involved in interferon signaling and have been suggested in numerous COVID-19 studies [39,40,41,42,43]. These results suggest that the network boosting of original GWAS signals can predict the host genes that are highly likely involved in COVID-19.

## 4. Discussion

In this paper, we presented COVID-GWAB, a web server-based tool that enables the discovery of new candidate genes related to COVID-19 from GWAS summary statistics. COVID-GWAB utilizes the human functional gene network, HumanNet [11], to boost GWAS data based on their connections to co-functional genes on the network. Although GWAS provides useful information related to many diseases within the population, there can be a loss of connection between the genotypes and the disease phenotypes due to limited sample sizes, stringent thresholds, and difficulties in interpretation. Network-based boosting can provide more biologically relevant results with its highly confident and large-scale edges connecting the functional genes.

To validate the boosting results, we compared the results of COVID-GWAB and GWAS without boosting using the COVID-19 geneset library and single-cell transcriptome datasets. With these datasets, we interpreted the biological relevance and interpretability of the COVID-GWAB results. The COVID-GWAB results, especially those genes that were newly discovered through boosting, showed higher appearance frequencies throughout numerous COVID-19 experiments as well as better overlaps with the DEGs from the single-cell datasets. Furthermore, the results reflected both known COVID-19-related genes and new gene candidates that are currently being studied throughout the various literature. In conclusion, COVID-GWAB provides an easy-to-use web server for exploring COVID-19 GWAS data, with various summary and validation tools.

## Data Availability

The web server is available at https://inetbio.org/covidgwab/ (accessed on 2 September 2022).

## Supplementary Materials

The following supporting information can be downloaded at: https://www.mdpi.com/article/10.3390/biom12101446/s1, Table S1: Single-cell RNA-seq datasets used for COVID-GWAB validation; Table S2: Top GWAB predictions with Release 6 of COVID-19 HGI GWAS data (B2).
