# Supplementary material for: COVID-GWAB: A Web-Based Prediction of COVID-19 Host Genes via Network Boosting of Genome-Wide Association Data
Source: Biomolecules. 2022 Oct 9;12(10):1446. doi: 10.3390/biom12101446 (PMC9599684; doi:10.3390/biom12101446)
Supplement: Supplementary file 1 [file biomolecules-12-01446-s001.zip › biomolecules-1924495-supplementary.pdf]

Supplementary Information

COVID-GWAB: a web-based prediction of COVID-19 host genes via network boosting of genome-wide association data

Seungbyn Baek, Sunmo Yang, and Insuk Lee

Supplementary Table S1. Single-cell RNA-seq datasets used for COVID-GWAB validation

Supplementary Table S2. Top GWAB predictions with Release 6 of COVID-19 HGI GWAS data (B2).

Supplementary Table S1. Single-cell RNA-seq datasets used for COVID-GWAB validation

Dataset	Tissue	Source	COVID	Control
GSE145926_BALF_10X	BALF	M. Liao *et al., Nat Med* (2020)	9	4
EGAS4571_PBMC_10X	PBMC	J. Schulte-Schrepping *et al., Cell* (2020)	27	22
EGAS4571_PBMC_Rhap	PBMC		50	13
EGAS4571_WB_Rhap	Whole Blood		17	16
GSE158055_PBMC_10X	PBMC	X. Ren *et al., Cell* (2021)	152	20
EGAS5465_PBMC_10X	PBMC	E. Stephenson *et al., Nat Med* (2021)	90	24
GSE171524_Lung_10X	Lung	J. Melms *et al., Nature* (2021)	19	7

Supplementary Table S2. Top GWAB predictions with Release 6 of COVID-19 HGI GWAS data (B2).

Gene	GWAB Score	GWAS Score	GWAS significance	GWAB rank	GWAS rank	Library Freq.	Rank Diff	Between centrality	Degree centrality	Category
CCR9	92.73512	88.21526	sig	1	1	8	0	102	19	Chemokine receptors
CXCR6	52.47783	48.105	sig	5	5	5	0	11	18	Chemokine receptors
CCR1	40.08533	34.90946	sig	7	7	54	0	340	19	Chemokine receptors
CCR3	38.40936	34.90946	sig	8	7	16	-1	11	17	Chemokine receptors
CCR5	28.17372	24.3163	sig	9	9	51	0	11	17	Chemokine receptors
CCR2	28.13037	24.3163	sig	10	9	25	-1	98	18	Chemokine receptors
HNRNPL	16.66227	3.405287	insig	21	3166	8	3145	1566	22	RNA-protein interactome
APP	16.14434	3.161768	insig	24	4621	26	4597	2944	24	Neurodegenerative Disease
LRRK2	16.07606	3.527185	insig	25	2629	20	2604	1425	20	Neurodegenerative Disease
NTRK1	14.07656	2.588768	insig	29	10495	14	10466	837	16	Kinase
STAT1	13.89319	1.622202	insig	32	21435	136	21403	654	16	interferon
ESR2	13.77369	2.784177	insig	33	8117	6	8084	1377	20	interaction with ACE2
TRIM25	13.67487	2.091912	insig	34	17506	80	17472	2094	23	innate immunity
IRF9	13.45149	3.561173	insig	36	2455	91	2419	213	12	Interferon axis
STAT2	12.87742	2.347502	insig	38	14012	89	13974	702	16	RNA-protein interactome, interferon
MOV10	12.20608	2.137261	insig	41	16917	44	16876	1642	19	RNA-protein interactome
CXCR4	11.9392	3.595765	insig	42	2337	35	2295	787	22	non chr3 chemokine Receptor
CCL5	11.85323	3.096812	insig	43	5142	47	5099	146	19	non chr3 chemokine
EGLN3	11.76585	2.770011	insig	45	8284	12	8239	979	17	Hypoxia inducible factor
FYN	11.75447	3.269282	insig	46	3902	17	3856	486	13	Kinase
SRC	11.31846	2.704631	insig	56	8965	52	8909	829	17	Kinase
ABL1	10.62772	2.861098	insig	91	7261	19	7170	304	12	Kinase
CXCL9	10.47801	3.254168	insig	96	3981	39	3885	300	21	non chr3 chemokine
EGFR	9.761761	2.063879	insig	134	17798	40	17664	NA	NA	RNA-protein interactome
ANXA1	8.416143	2.465492	insig	302	12269	39	11967	NA	NA	RNA-protein interactome
